# The TM6SF2 E167K genetic variant induces lipid biosynthesis and reduces apolipoprotein B secretion in human hepatic 3D spheroids

**DOI:** 10.1038/s41598-019-47737-w

**Published:** 2019-08-12

**Authors:** Sebastian Prill, Andrea Caddeo, Guido Baselli, Oveis Jamialahmadi, Paola Dongiovanni, Raffaela Rametta, Kajsa P. Kanebratt, Arturo Pujia, Piero Pingitore, Rosellina Margherita Mancina, Daniel Lindén, Carl Whatling, Annika Janefeldt, Mikael Kozyra, Magnus Ingelman-Sundberg, Luca Valenti, Tommy B. Andersson, Stefano Romeo

**Affiliations:** 1 DMPK, Cardiovascular, Renal and Metabolism, IMED Biotech Unit, AstraZeneca, Gothenburg, Sweden; 2 Department of Molecular and Clinical Medicine, University of Gothenburg, Gothenburg, Sweden; 3 Internal Medicine and Metabolic Diseases, Fondazione IRCCS Ca’ Granda Ospedale Maggiore Policlinico Milano, Department of Pathophysiology and Transplantation, Università degli Studi di Milano, Milan, Italy; 4 Clinical Nutrition Unit, Department of Medical and Surgical Sciences, University Magna Graecia, Catanzaro, Italy; 5 Bioscience Diabetes, Cardiovascular, Renal and Metabolism, IMED Biotech Unit, AstraZeneca, Gothenburg, Sweden; 6 Division of Endocrinology, Department of Neuroscience and Physiology, Sahlgrenska Academy, University of Gothenburg, Gothenburg, Sweden; 7 Translational Sciences, Cardiovascular, Renal and Metabolism, IMED Biotech Unit, AstraZeneca, Gothenburg, Sweden; 8 Department of Physiology and Pharmacology, Section of Pharmacogenetics, Karolinska Institutet, Stockholm, Sweden; 9 Cardiology Department, Sahlgrenska University Hospital, Gothenburg, Sweden

**Keywords:** Medical genomics, Tissue engineering, Target validation, Non-alcoholic fatty liver disease

## Abstract

There is a high unmet need for developing treatments for nonalcoholic fatty liver disease (NAFLD), for which there are no approved drugs today. Here, we used a human *in vitro* disease model to understand mechanisms linked to genetic risk variants associated with NAFLD. The model is based on 3D spheroids from primary human hepatocytes from five different donors. Across these donors, we observed highly reproducible differences in the extent of steatosis induction, demonstrating that inter-donor variability is reflected in the *in vitro* model. Importantly, our data indicates that the genetic variant *TM6SF2* E167K, previously associated with increased risk for NAFLD, induces increased hepatocyte fat content by reducing APOB particle secretion. Finally, differences in gene expression pathways involved in cholesterol, fatty acid and glucose metabolism between wild type and *TM6SF2* E167K mutation carriers (N = 125) were confirmed in the *in vitro* model. Our data suggest that the 3D *in vitro* spheroids can be used to investigate the mechanisms underlying the association of human genetic variants associated with NAFLD. This model may also be suitable to discover new treatments against NAFLD.

## Introduction

Nonalcoholic fatty liver disease (NAFLD) is considered a global epidemic^1^ and the most common cause of chronic liver disease worldwide, with increasing prevalence^2,3^. It comprises a range of conditions, from relatively benign simple steatosis to more progressed forms, including nonalcoholic steatohepatitis (NASH) and liver fibrosis which may progress to cirrhosis and hepatocellular carcinoma^4–6^ as well as cardiovascular morbidities^7,8^. NAFLD has a strong genetic component^9^, with the patatin-like phospholipase domain containing 3 I148M (rs738409, *PNPLA3* I148M) and transmembrane 6 superfamily member 2 E167K (rs58542926, *TM6SF2* E167K) genetic variants having the largest effect on the susceptibility to the entire spectrum of liver disease^9^.

Accumulation of intrahepatic fat is the hallmark of NAFLD. Despite a Mendelian Randomization study^10^ suggesting a causal role of fat accumulation in causing liver fibrosis, the mechanism behind the toxicity of intracellular neutral lipid is not clear. The increase in hepatocyte intracellular fat can be attributed to derangements of at least four main metabolic pathways: increased triglyceride (TG) synthesis, increased TG uptake, decreased fat secretion or decreased fatty acid (FA) oxidation^11^. In genetic association studies carriers of the *TM6SF2* E167K variant have substantially higher risk of NAFLD and at the same time lower risk for cardiovascular disease^12–14^. Human *TM6SF2* E167K carriers have lower circulating apolipoprotein B100 (APOB) levels^12,15^ suggesting a reduction in the number of secreted lipoproteins. The *TM6SF2* E167K genetic variant may result in a misfolded protein that is prone to intracellular degradation resulting in reduced protein levels^13,16^. *Tm6sf2* inactivation in mice is, like in humans, associated with hypocholesterolemia and increased liver fat accumulation. In addition, hepatic VLDL-TG secretion rate is reduced while the APOB secretion is unaffected in the *Tm6sf2* knock-out (KO) mice, indicating no change in the number of secreted lipoprotein particles from the liver^17^ in discordance with the human data^12,15^.

At present, there is no effective and approved pharmacotherapy available for the treatment of NAFLD. For development efforts aiming at novel treatments, disease models reflecting human NAFLD are required. Numerous dietary and genetic animal models are used for investigating the disease and its progression, of which most are rodent models^18,19^. The indisputable advantage of *in vivo* models is the representation of the (patho)physiological interplay of organs in the body. However, animal models have also clear limitations such as interspecies differences across liver pathways^20,21^, disease characteristics^21,22^, high costs and ethical concerns. Thus, human *in vitro* systems are commonly used to complement animal experimentation^23^. To address the limitations of simple human *in vitro* models culturing primary hepatocytes in a classical 2D monolayer, 3D spheroids generated from primary human hepatocytes have proven to be excellent surrogates for human physiology^24^ by providing an *in vivo*-like cellular microenvironment^24,25^. However, spheroids composed of primary human hepatocytes have not been exploited as a model to investigate molecular genetic mechanisms involved in the development of NAFLD.

Aim of this work was to generate a 3D spheroid model to study molecular genetics of NAFLD. To achieve this goal we first examined differences in hepatic lipid metabolism between wild type (WT) and carriers of the *TM6SF2* E167K genetic variant using human data from hepatic transcriptome. Next, we used a recent *in vitro* 3D spheroid NAFLD model from primary human hepatocytes^26^. This *in vitro* system reflects the liver physiology of WT and *TM6SF2* E167K donors.

## Results

### Enrichment analyses between *TM6SF2* wild type and *TM6SF2* E167K (rs58542926) mutant carriers

We examined liver-derived RNA-Seq data from 125 patients at risk for NAFLD diagnosed by liver biopsies (Supplementary Tables S1 and S2). Among these, 116 were wild type (WT) and 9 were E167K mutant carriers. Since DESeq2 analysis did not show any differentially expressed gene, we hypothesized that the insignificant adjusted p-values are the direct consequence of low number of heterozygous carriers of the *TM6SF2* E167K mutation (N = 9) compared to WT (N = 116). Therefore, to understand the metabolic changes in *TM6SF2* E167K carriers, we performed enrichment for genes with a nominal p-value < 0.05 which are related to hepatic glucose and lipid metabolism using Gene Ontology (GO) biological process (BP) terms and KEGG biological pathways (Supplementary Table S4). The most enriched GO terms and KEGG pathways were those related to cholesterol (cholesterol - GO:0006695 and sterol biosynthetic - GO:0016126 processes in GO-BP, and terpenoid backbone biosynthesis in KEGG), TG (GO:0006641) and FA metabolism (GO:0019217). Interestingly, KEGG pathways associated with viral hepatitis (Hepatitis C and B) and NAFLD were significantly enriched in our list of examined genes.

### Spheroid morphology

**Figure 1:**
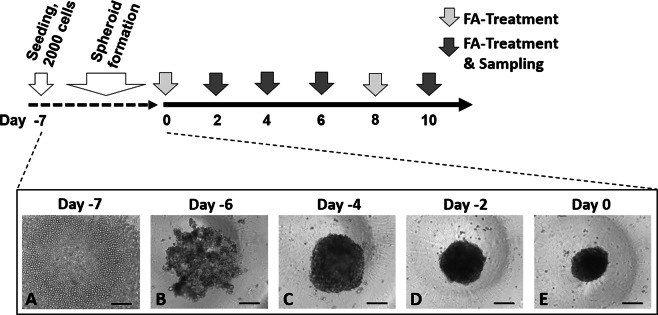
Overview of spheroid generation and study design. Spheroids were generated by incubating 2000 primary human hepatocytes for 7 days in 384- or 96-well ultra-low attachment plates. Initiation of spontaneous cell aggregation was observed within one day after seeding. Hepatocytes from five individual donors were used in two independent experiments for WT-1, WT-2 and *TM6SF2* E67K-2, and in one independent experiment for WT-3 and *TM6SF2* E167K-1, respectively (data from repeat experiments shown in Supplementary Fig. S7). For each experiment, an individual vial of cryopreserved primary hepatocytes was used for spheroid preparation. Bright-field images representatively show spheroid formation by spontaneous aggregation of hepatocytes obtained from donor WT-1. Hepatocytes from all other donors showed similar spheroid formation behavior, yielding fully formed spheroids after 7 days. Arrows in the timeline depict medium changes for incubation with FA and samplings for bright field (Figs 2A–C and S3) and confocal (Figs 5B and S4) imaging, intracellular ATP content (Fig. 3), intracellular and secreted APOB (Figs 4 and 7A,B), intracellular total TG content (Fig. 5A) and gene expression profiling (Figs 6 and 7C), respectively. Bars = 200 µm. Abbreviations: ATP, adenosine triphosphate; APOB, APOLIPOPROTEINB100; FA, fatty acid; TG, triglyceride, WT, wild type.

Next, we generated a 3D spheroid model from primary human hepatocytes obtained from five individual donors, of which two were heterozygous for the *TM6SF2* E167K allele, and three were WT. To avoid confounders, we refrained from using primary human hepatocytes carrying the *PNPLA3* rs738409 variant (encoding for an isoleucine to methionine substitution at position 148 of the PNPLA3 protein, I148M), the strongest common genetic variant increasing liver fat content^27^. Furthermore, donors were genotyped for the PNPLA3 rs2294918 (E434K), GCKR rs1260326 (P446L) and MBOAT7 rs641738 (C > T) NAFLD risk alleles (Supplementary Table S5). A total of 2,000 cryopreserved primary human hepatocytes from each of the five donors were seeded per well at day −7 (Fig. 1A). After one day cells started to aggregate, forming a 3D structure (Fig. 1B). After three days of culture the aggregates resulted in spheroidal structures (Fig. 1C), further condensing over the following 4 days (Fig. 1D,E). During the spheroid formation the diameter of the entirety of seeded cells from the WT-1 donor decreased by approximately 45%, starting from a diameter of 450 µm (Supplementary Fig. S1). This formation pattern was similar in all donors. At day 0 the spheroids were fully formed with intact and distinct borders (Fig. 1E).

### Spheroids are viable and well differentiated up to 10 days with and without fatty acid incubation

**Figure 2:**
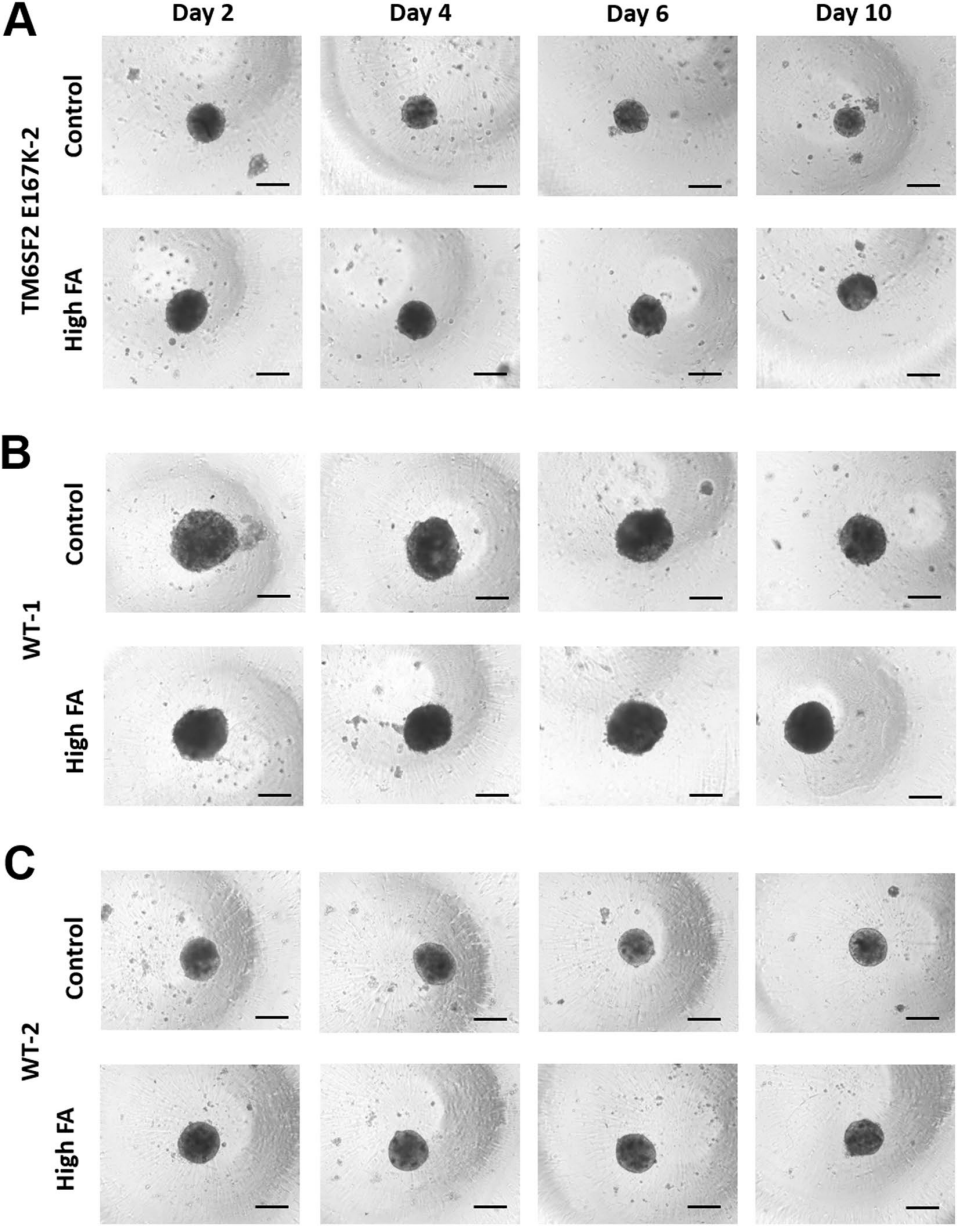
Spheroids show no morphological changes for up to 10 days of FA treatment. Bright-field images of spheroids from the *TM6SF2* E167K-2 (**A**), WT-1 (**B**) and WT-2 (**C**) donor, without (top row, Control, FA-free BSA) and with FA treatment (bottom row, high FA, BSA + 213 µM OA/107 µM PA) for up to 10 days showed no morphological differences between the two populations. Corresponding bright-field images of the *TM6SF2* E167K-1 and WT-3 donors are shown in Supplementary Fig. S3. Bar = 200 µm. Abbreviations: BSA, bovine serum albumin; FA, fatty acid; OA, oleic acid; PA, palmitic acid; WT, wild type.

To generate a 3D spheroid hepatocyte model of NAFLD, we incubated spheroids with increasing amounts of 2:1 oleic:palmitic acid-enriched medium (160 µM, 240 µM (data not shown) and 320 µM total FA concentration, conjugated to bovine serum albumin (BSA), as described previously^26^. Medium was changed every two days with sampling of spheroids and medium supernatant as depicted in Fig. 1. To test whether FA incubation results in spheroid morphological changes we measured the size at treatment days two, four, six and ten. We found no differences in diameter across days and with different treatment concentrations of FAs (Supplementary Fig. S2). We also examined shape and spheroid borders, which remained spherical with intact and distinct borders throughout all treatment conditions and time points (Fig. 2, Supplementary Fig. S3).

**Figure 3:**
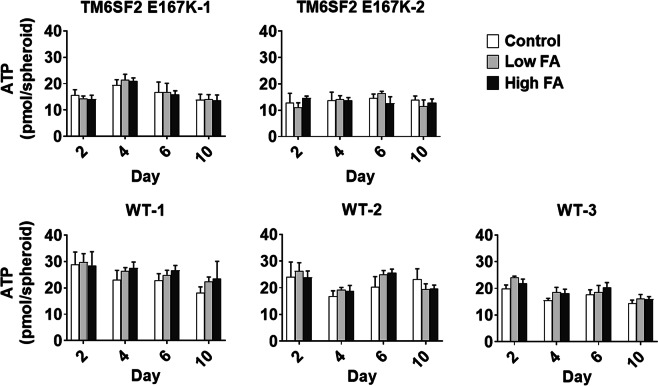
Spheroid viability is not compromised by FA treatment. Total intracellular ATP content measured by luminescence using CellTiter-Glo® 3D Cell Viability Assay (Promega) in primary human hepatocyte spheroids with and without FA treatment for up to 10 days, using control (FA-free BSA) and two FA concentrations (low FA, BSA + 107 µM OA/53 µM PA and high FA, BSA + 213 µM OA/107 µM PA). Spheroids were generated from hepatocytes derived from five individual donors, out of which two were mutant and three were wild type for *TM6SF2* E167K. Data are mean ± SD of eight single spheroid replicates per time point and condition and from one spheroid preparation per donor. Abbreviations: ATP, adenosine triphosphate; BSA, bovine serum albumin; FA, fatty acid; OA, oleic acid; PA, palmitic acid.

To assess the viability of cultured spheroids, we determined intracellular ATP content after incubation with and without FAs using two different concentrations at treatment day two, four, six and ten (Fig. 3). We observed no differences in the ATP concentration among groups throughout treatment concentrations and time points.

**Figure 4:**
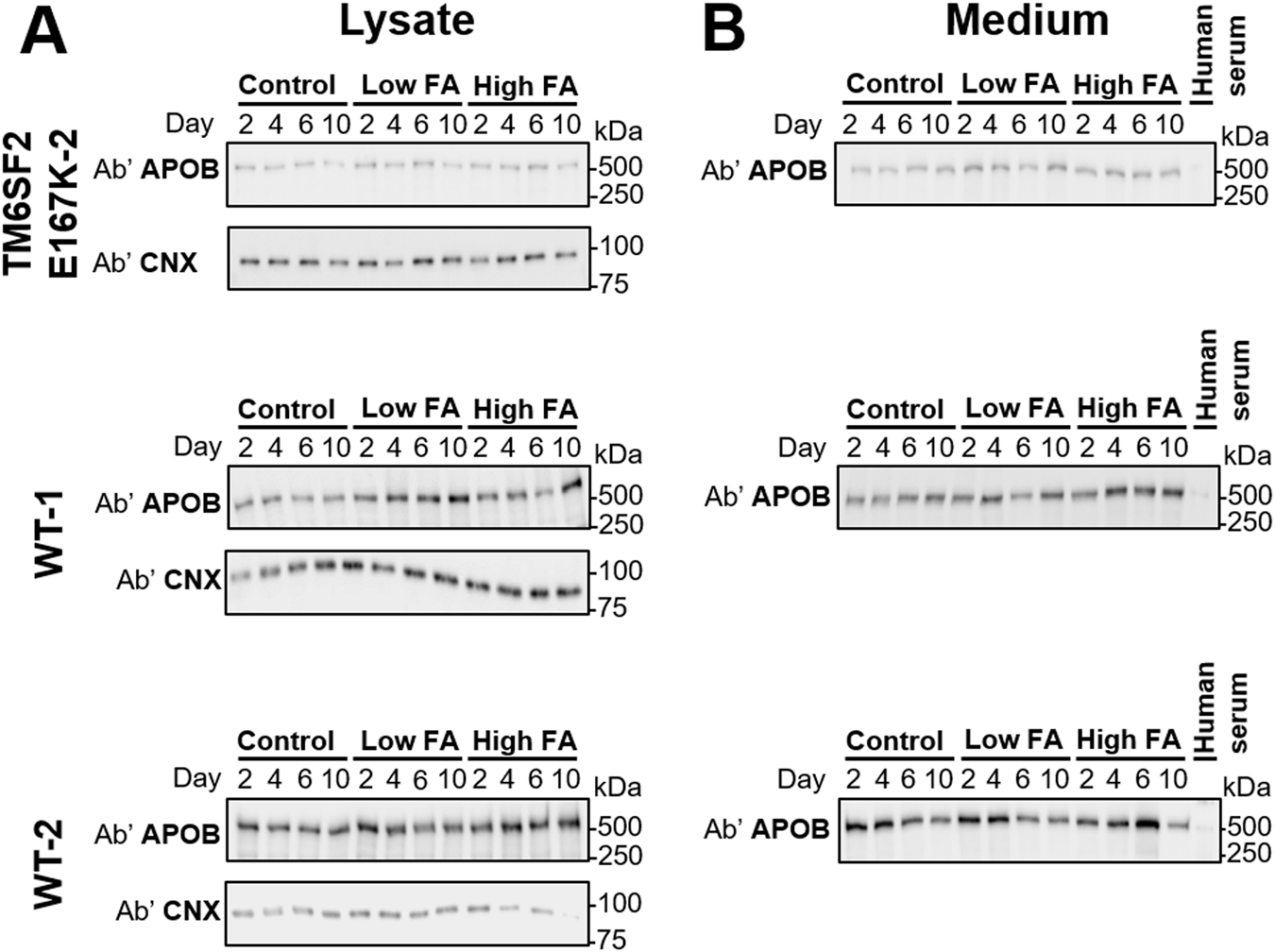
Spheroids are well-differentiated for up to 10 days of fatty acid incubation. Intracelluar (**A**) and extracellular (**B**) APOB protein levels in spheroids and culture medium, respectively, up to 10 days of FA incubation. APOB was measured by immunoblotting analysis, using anti-APOB and anti-calnexin (CNX) antibodies, from cell lysate and medium supernatant of primary human hepatocyte spheroids that were derived from three individual donors. APOB levels were assessed with low (BSA + 107 µM OA/53 µM PA), high (BSA + 213 µM OA/107 µM PA) and without (FA-free BSA) incubation with FAs. Original, uncropped blots are presented in Supplementary Fig. S8. This figure shows data from of one independent experiment per donor. Abbreviations: Ab’, antibody; APOB, APOLIPOPROTEINB100; BSA, bovine serum albumin; CNX, Calnexin; FA, fatty acid.

Next, we evaluated differentiation of hepatocytes in spheroids by determining intracellular and extracellular levels of APOB, a protein only expressed in hepatocytes and involved in hepatocyte TG secretion by very low-density lipoprotein (VLDL), a pathway present exclusively in hepatocytes. We found that APOB protein synthesis was preserved (Figs 4A and 7A,B) from beginning of treatment up to the end (10 days). Also, the synthesized APOB was consistently secreted into the extracellular space up to the end of the treatment, as shown by protein levels in the medium (Figs 4B and 7A,B).

All these data suggest that spheroids are viable, well differentiated and functional up to 10 days of incubation with FAs.

### FA incubation results effectively in intracellular neutral lipid accumulation

**Figure 5:**
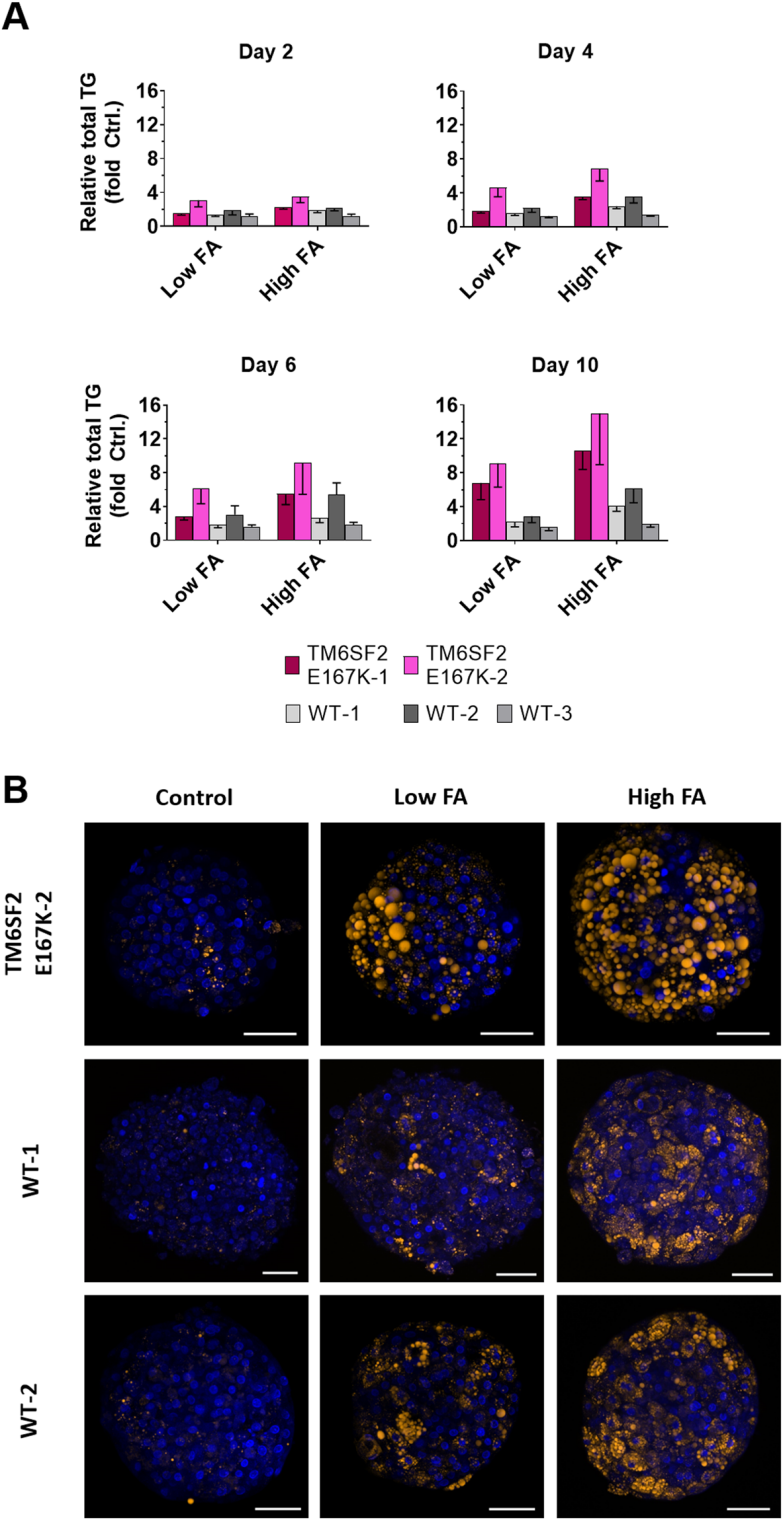
Spheroids accumulate intracellular neutral triglycerides upon incubation with fatty acids. Spheroids were incubated with a 2:1 mixture of OA and PA (conjugated to BSA), for up to 10 days. Exposure was conducted using low (BSA + 107 µM OA/53 µM PA) and high (BSA + 213 µM OA/107 µM PA) FA concentration, as well as FA-free BSA (control). (**A**) Total TG content of liver spheroids, measured by fluorescence intensity using AdipoRed™ Assay Reagent. Spheroids were formed using primary human hepatocytes derived from five individual donors, out of which two were mutant (*TM6SF2* E167K-1, *TM6SF2* E167K-2) and three were wild type (WT-1, WT-2, WT-3) for *TM6SF2* E167K. TG content is shown as fold change relative to vehicle control (FA-free BSA). Data represent mean values ± SD from 16 individual spheroid replicates per time point and condition, from one spheroid preparation per donor (repeat experiments of the donors *TM6SF2* E167K-2, WT-1, and WT-2 are shown in Supplementary Fig. S7). (**B**) Nile red staining of low FA and high FA-incubated liver spheroids derived from three individual hepatocyte donors, after 10 days of FA incubation. Bars = 50 µm. Abbreviations: BSA, bovine serum albumin; FA, fatty acid; OA, oleic acid; PA, palmitic acid; TG, triglyceride.

To test whether FA incubation results in an increased fat content in the spheroids, we measured total intracellular TG content in single cell suspension obtained from disintegrated spheroids by fluorescence intensity using AdipoRed™ assay reagent (Fig. 5A) and by confocal Nile Red imaging of fixed and morphologically intact spheroids after two, six (Supplementary Fig. S4) and 10 days (Fig. 5B) of FA incubation. We found an increase in the total amount of intracellular fat content after incubation with increasing amounts of FAs in all donors (Supplementary Fig. S5,) and with increasing duration of the incubation (Supplementary Fig. S6). Incubation with an intermediate concentration of FA (240 µM; 160 µM OA/80 µM PA) led to an intermediate treatment response lying between the ones of low and high FA concentration (data not shown). Overall, the TM6SF2 E167K-1 and -2 donors had higher intracellular lipids from day four to the end of the incubation.

Importantly, donor-specific patterns in fat accumulation were robustly confirmed during one repeat experiment for donors *TM6SF2* E167K-2, WT-1 and WT-2, using low and high FA concentrations (Supplementary Fig. S7).

### RNA measurement of key metabolic genes from human genetic analyses

Next, to compare the *in vitro* results with our *ex vivo* data in human liver, we measured the gene expression levels of the enzymes in the cholesterol, FA metabolism and gluconeogenesis pathway that were significantly differentially expressed in humans carrying the TM6SF2 mutant protein, both in spheroids carrying the *TM6SF2* E167K and WT allele. We compared the expression levels in *TM6SF2* E167K (pooled from 2 donors) and WT (pooled from 3 donors) spheroids after 2 days of incubation with high FA concentration (320 µM total FA, 213 µM OA/107 µM PA), and examined the directional regulation (up or down) of these genes (Supplementary Table S3).

**Figure 6:**
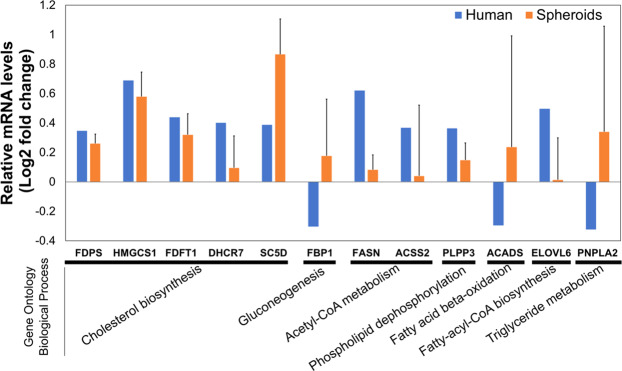
Transcriptional data derived from human liver and primary human hepatocyte spheroids show consistent changes in metabolic pathways when the *TM6SF2* E167K variant is carried. Genes related to cholesterol biosynthesis, de novo lipogenisis and phospholipid dephosphorylation were upregulated both in human livers and spheroids carrying the rs58542926 risk variant and exposed for 2 days to high FA concentration (BSA + 213 µM OA/107 µM PA). To be comparable to RNA-Seq analysis, the regulation of key genes examined in RT-PCR were evaluated relative to FA-treated versus FA-free BSA control samples for both *TM6SF2* E167K and WT. The reported values are the log2 normalized fold changes (log2 FC^MUT^/FC^WT^). Error bars represent the standard error of the mean (SEM) from the two *TM6SF2* E167K carriers and the three WTs. Genes involved in Gluconeogenisis (FBP1), FA β-oxidation (ACADS), and TG metabolism (PNPLA2) where differentially regulated in humans and spheroids carrying the *TM6SF2* E167K risk variant.

Consistent with the human *ex vivo* data, genes related to cholesterol biosynthesis (FDPS, HMGCS1, FDFT1, DHCR7 and SC5D), *de novo* lipogenesis (FASN and ACSS2) and phospholipid dephosphorylation (PLPP3) were upregulated in the hepatic *TM6SF2* E167K spheroids, while gluconeogenesis (FBP1) was upregulated in the spheroids and downregulated in humans carrying the E167K risk variant (Fig. 6). ACADS, an enzyme involved in FA β-oxidation was upregulated in spheroids, but downregulated in human *ex vivo* data. PNPLA2, an enzyme involved in TG metabolism, was upregulated in spheroids, but downregulated in humans (Fig. 6).

### Spheroids carrying the *TM6SF2* E167K variant have lower APOB secretion

**Figure 7:**
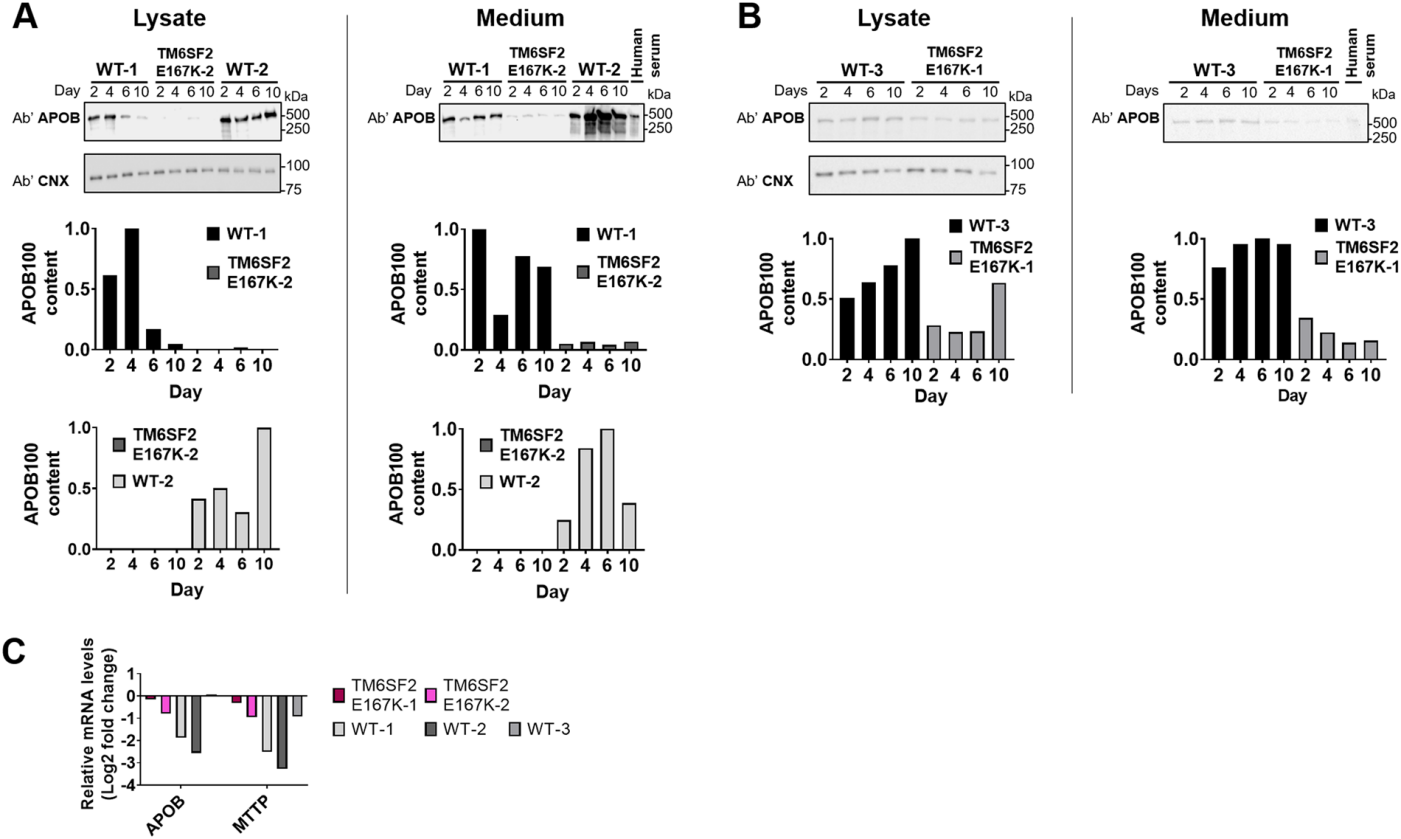
Liver spheroids derived from the *TM6SF2* E167K donors show substantially decreased synthesis and secretion of APOB as compared to spheroids derived from the WT donors. (**A**) Immunoblots using anti-APOB and anti-calnexin (CNX) antibodies comparing APOB synthesis (lysate) and secretion (medium) in WT-1, *TM6SF2* E167K-2 and WT-2 show decreased APOB particle number intracellularly and in medium samples obtained from the *TM6SF2* E167K-2 donor after incubation with high FA (BSA + 213 µM OA/107 µM PA) for up to 10 days. Quantifications relative to WT-1 and WT-2 are shown below the cropped blots. Original, uncropped blots are provided in Supplementary Fig. S9A. (**B**) Immunoblot and quantifications layout as presented in (**A**), showing donor WT-3 vs. *TM6SF2* E167K-1. Original, uncropped Blots are provided in Supplementary Fig. S9B. (**C**) Relative mRNA levels of APOB and MTTP (log2 transformed) after a 2-day incubation with high FA (BSA + 213 µM OA/107 µM PA), normalized to GAPDH and expressed relative to the levels of FA-free BSA treated control. APOB and MTTP are slightly downregulated in spheroids derived from the *TM6SF2* E167K donors, while the WT-1 and WT-2 donors show a stronger downregulation of APOB and MTTP. In WT-3, APOB expression was unchanged and MTTP slightly downregulated. The figure represents data from one experiment per donor and RNA was extracted from 20 pooled spheroids per donor. Abbreviations: Ab’, antibody; APOB, APOLIPOPROTEINB100; CNX, calnexin; FA, fatty acid; MTTP, microsomal triglyceride transfer protein; OA, oleic acid; PA, palmitic acid.

To understand the consequences of carriage of the *TM6SF2* E167K variant, we examined the relative difference in APOB levels in the medium supernatant from spheroids of the donors. Medium obtained from *TM6SF2* E167K spheroids contained lower levels of APOB (Fig. 7A,B). Since there is one APOB protein per secreted VLDL particle^28^, these data indicate that hepatocyte spheroids from *TM6SF2* E167K carriers secrete fewer APOB VLDL particles compared to WT spheroids. To further understand whether the reduction in the APOB was due to changes in *APOB* expression we examined mRNA levels of *APOB* at day 2 of incubation with FAs in spheroids from both types of donors. We observed a reduction of mRNA levels in four out of the five donors, where the donors carrying the *TM6SF2* E167K protein had a slightly lower reduction (−0.15 fold, *TM6SF2* E167K-1 and −0.79 fold, *TM6SF2* E167K-2) than the WT-1 (−1.87 fold) and WT-2 donor (−2.56 fold). APOB expression in WT-3 was unchanged (Fig. 7C). We also did not observe any difference in *APOB* expression, nor in the expression of microsomal triglyceride transfer protein (MTTP), a protein necessary for the assembly of APOB-containing lipoproteins, in the transcriptome of patients at risk for NAFLD carrying the TM6SF2 E167K protein.

## Discussion

In this work we show in both human liver tissue samples and in 3D spheroid cultures of primary human hepatocytes that carriage of the TM6SF2 E167K mutant protein is associated with an upregulation of the cholesterol and FA biosynthesis pathways. We also demonstrate that spheroid cultures with human primary hepatocytes obtained from two *TM6SF2* E167K donors have lower secretion of APOB compared to spheroids formed from hepatocytes of three wild type (WT) hepatocyte donors investigated.

We started by examining enrichment for significantly differentially expressed genes (by GO and KEGG pathways analyses) in human liver from individuals at risk for liver disease carrying the *TM6SF2* E167K sequence variant, as compared to the WT. We found a striking upregulation of most of the enzymes involved in lipid synthesis and specifically of cholesterol and FA metabolism with both GO and KEGG. Interestingly, KEGG analyses found an enrichment of genes which are classified as involved in the pathogenesis of viral hepatitis and NAFLD. This is in line with the human genetic association data showing that *TM6SF2* E167K carriers have higher risk not only for NAFLD but also for progression of liver damage in patients infected by viral hepatitis B and C^29,30^.

Next, we used a novel 3D spheroid NAFLD model utilizing primary human hepatocytes^24^ from five individual donors, two carrying the TM6SF2 E167K protein and three the WT protein. In both *TM6SF2* E167K and WT the ATP levels, as well as APOB synthesis and secretion were preserved up to ten days after formation, indicating that spheroids were viable, well differentiated and functional within this time frame. Next, to induce intracellular lipid accumulation, we exposed the spheroids to increasing doses of a mixture of saturated and monounsaturated FAs for up to 10 days. FA load did not affect spheroid viability and differentiation. Most importantly, in the spheroids derived from the *TM6SF2* E167K donor we consistently found a larger increase in the intracellular fat content as compared to the WTs, which was dose- and time-dependent. This suggests that the spheroids may represent a reliable model to study the mechanism underpinning predisposition to lipid accumulation in carriers of the *TM6SF2* E167K variant. Furthermore, the phenotypic expression of the mutation in the presence of excess FAs is consistent with the recent gene environment interaction observed for this gene variant^31^.

We examined the intracellular biological pathways by differential expression analysis and found that, consistent with human data, *TM6SF2* E167K spheroids had upregulated genes involved in the cholesterol, FA and glucose metabolism. Overall, the directional regulation of genes involved in cholesterol metabolism was consistent in humans and the spheroid model. We found a discordance in enzymes involved in lipolysis (PNPLA2), gluconeogenesis (FBP1) and β-oxidation (ACADS). While PNPLA2 is a key enzyme in lipolysis, we did not observe any significant change in the expression of other lipolytic enzymes (LIPE and MGLL) involved in TG hydrolysis in humans.

Taken all this together, our data indicate that the spheroids generated from *TM6SF2* E167K primary hepatocytes have similar metabolic changes present as in livers of human carriers. These changes, namely increased cholesterol and FA biosynthesis, may contribute to the increase in intracellular fat. Interestingly, excess in free cholesterol is known to cause hepatic damage^32,33^. Our data are consistent with increased cholesterol esters in the liver of *TM6SF2* E167K carriers^34^. Therefore, although we could not evaluate protein expression and directly measure *de novo* lipid synthesis, our data suggest that the progression of NAFLD in *TM6SF2* E167K carriers could be ascribed not only to reduced lipid secretion^13^, but also to the excess synthesis of cholesterol or other lipotoxic lipid species^32,33^. Interestingly, the mouse knockout (KO) model and *in vitro* overexpression of the *TM6SF2* E167K sequence variant accumulates intrahepatic fat, but this was associated with reduced cholesterol biosynthesis^17,35^, possibly suggesting that the mouse model may not closely mirror human physiology. However, in contrast to our results, a previous study reported downregulation of lipid synthesis in the liver of *TM6SF2 E167K* carriers^34^.

Next, to investigate on the mechanism of intracellular fat retention we examined the APOB secretion as well as intracellular synthesis in spheroids. We found that *TM6SF2 E167K* spheroids show a lower secretion of APOB, coupled to lower intracellular APOB protein levels. The *Tm6sf2* KO mouse was found to have unchanged secretion of APOB particle number^17^, while a human genetic association study showed lower levels of circulating APOB^15^. Our data are consistent with the human genetic association study, indicating a defect in secretion of APOB particles, which may cause intracellular lipid increase. Regarding the cause of reduction in APOB secretion, we found lower intracellular levels of APOB. This could be due to an increase in APOB degradation, possibly due to unlipidated or underlipidated forms of APOB^36^. These data are consistent with the notion that decreased transfer of FAs on nascent VLDL in the endoplasmic reticulum (lipidation) contributes to intrahepatic fat accumulation in *TM6SF2 E167K* carriers^17^. However, differently than in the mice model, we showed that in human hepatocytes this defect also results in decreased APOB protein levels and secretion. A limitation of our study is that we cannot take into account the effect of intestinal uptake of lipids in chylomicrons which is also reduced in the presence of the *TM6SF2* mutant protein in the postprandial state^37^. However, the data presented in this study and in previous genetic studies were in a fasting state, minimizing the interference of lipoproteins of the fed state^12,15^.

The main limitation of our study is that the number of mutant donors was limited and therefore the results should be interpreted with caution. In our screening efforts, it was not possible to identify further *TM6SF2* E167K hepatocyte donors because of the relatively low allele frequency of this sequence variant. It should also be taken into consideration that the two mutant donors were of younger age (13 and 22 years, Supplementary Table S5) as compared to the three wild type donors (30, 44 and 78 years, Supplementary Table S5), which could influence hepatocyte function.

In conclusion, we showed a lower APOB secretion in primary human hepatocyte spheroids from *TM6SF2* E167K as compared with WT, while the cholesterol biosynthesis was higher in the *TM6SF2* E167K spheroids when exposed to FAs. These data, generated in an experimentally accessible *in vitro* setting, recapitulate the differences found in human WT and *TM6SF2* E167K carriers. Hence, 3D spheroids may represent a useful model to study mechanism of NAFLD development and impact of genetic background, offering a promising tool for drug development.

## Materials and Methods

### Materials

Unless stated otherwise, medium supplements and reagents were obtained from Sigma-Aldrich (Stockholm, Sweden) and LifeTechnologies (Stockholm, Sweden).

### Patient cohort, RNA seq and statistical analyses

RNASeq was performed in 125 severely obese individuals who underwent to percutaneous liver biopsy performed during bariatric surgery, as described in a previous study assessing the role of a genetic variant in the protein phosphatase 1 regulatory subunit 3B gene (*PPP1R3B*)^38^. Informed consent was obtained from each patient and the study protocol was approved by the Ethical Committee of the Fondazione IRCCS Ca’ Granda, Milan and conformed to the ethical guidelines of the 1975 Declaration of Helsinki. Individuals with increased alcohol intake (men: >30 g/day; women: >20 g/day), viral and autoimmune hepatitis or other causes of liver disease were excluded. Steatosis was graded based on the percentage of affected hepatocytes as 0: 0–4%, 1: 5–32%, 2: 33–65%, and 3: 66–100%. NAFLD Activity Score (NAS) was assessed by systematic evaluation of hepatocellular ballooning and necroinflammation. Fibrosis was also staged according to the recommendations of the NAFLD clinical research network^39^.

RNA extraction was performed using miRNeasy mini-kit (Qiagen, Hilden, Germany), according to the manufacturer’s instructions. Agilent 2100 Bioanalyzer was used for RNA qualitative assessment, high-quality samples (RNA integrity number >7) were used for library preparation. RNA was sequenced in paired-end mode using an Illumina HiSeq 4000 (Novogene, Hong Kong, China). Reads quality evaluation and trimming was conducted using FastQC (Babraham Bioinformatics, Cambridge, UK) and Trimmomatic^40^ packages. Reads were mapped against GRCh37 reference genome^41^ using STAR aligner^42^. Samples with insufficient mapping quality (<10 million mapped reads, uniquely mapped <60% mapped reads) were excluded. Per gene reads were counted according to the ENSEMBL human transcript reference assembly version 75 exploiting RSEM package^43^. Raw counts normalization and differential gene expression analysis were performed exploiting DESeq2 package^44^ according to the standard workflow. P-values were corrected for testing multiplicity by Benjamini-Hochberg false discovery rate^45^.

### Gene Ontology (GO) and pathway enrichment analysis

We used GeneTrail2 tool (26787660) to test for the enrichment of functional annotations among differentially expressed genes between *TM6SF2* rs58542926 wild type and E167K mutant carriers (nominal p-value < 0.05). The tests were carried out using Gene Ontology (GO) Biological Processes (BP) and KEGG pathways. Hypergeometric distribution was used to calculate p-values, which were subsequently adjusted for multiple hypothesis testing using Benjamini and Hochberg false discovery rate (FDR) correction^45^.

### 3D liver spheroids formation and maintenance

All cryopreserved primary human hepatocytes used in this study were obtained from BioIVT (Brussels, Belgium) and thawed according to the supplier’s instructions. It was not possible to identify additional *TM6SF2* E167K donors. The sourcing details and demographics of the donors are available in Supplementary Table S5. For spheroid formation, the methods described in Bell *et al*.^24^ and Kozyra *et al*.^26^ were mainly employed, using slight variations. In short, 2,000 viable cells per well were seeded into ultra-low adhesion 384-well plates (3830 Corning, Corning, NY, USA) or ultra-low adhesion 96-well plates (7007 Corning, Corning, NY, USA). Total viabilities of the cell preparations after thawing ranged between 90–95% for hepatocytes from all donors, except for the *TM6SF2* E167K-1 donor (84%). Since lower total cell viability is frequently associated with reduced spheroid formation capability, brief exposure to the pan-caspase apoptosis inhibitor Z-VAD-FMK was used to support spheroid formation, as described recently^46^. The same treatment was done for the WT-3 donor to allow for direct comparisons of both preparations (Fig. 7B). After seeding, the plates were centrifuged at 100 × *g* for 2 min. Cells were seeded in Williams E medium (PAN-Biotech, Aidenbach, Germany), supplemented with 5.5 mM glucose, 2 mM L-glutamine, 100 units/ml penicillin, 100 µg/ml streptomycin, 100 pM insulin, 5.5 µg/ml transferrin, 6.7 ng/ml sodium selenite, 100 nM dexamethasone and 10% fetal bovine serum. The cells were allowed to aggregate and form spheroids for 7 days, with daily medium change starting on the fourth day of culture. Medium changes were performed with FBS-free medium of the otherwise same formulation as described above (maintenance medium). After the start of fatty acid incubation, medium was changed every 48 hours (Fig. 1).

### Steatosis induction

Hepatic steatosis was induced by metabolic stimulation with the unsaturated FA oleic acid (OA) and the saturated FA palmitic acid (PA) in a ratio of 2:1, mimicking human plasma concentrations of free fatty acids (FFAs), as described previously^26^. Exposure started 7 days after seeding (regarded as day 0, *cf*. Fig. 1). FAs were complexed to bovine serum albumin (BSA) for facilitating FFA uptake by the hepatic spheroids. In short, complexing was achieved by incubating the FAs with 10% BSA (wt/vol, FA-free) in a molar ratio of 1:5 at 40 °C for 2 hours. All complexed solutions were sterile filtered. The OA/PA complex was added to maintenance medium (described above) for two final total FA concentrations: low, 160 µM (107 µM OA/53 µM PA) and high, 320 µM (213 µM OA/107 µM PA). Controls were supplemented with FA-free BSA in equimolar concentrations as used in high FA incubation.

### Adenosine triphosphate content

Total intracellular adenosine triphosphate (ATP) content was quantified by luminescence using CellTiter-Glo® 3D Cell Viability Assay (Promega, Madison, WI, USA) with a slightly modified protocol. In brief, for each replicate one individual spheroid was transferred by sedimentation into a white 96-well assay plate (3903 Corning, Corning, NY, USA) containing 50 µl PBS (w/o Mg^2+^, Ca^2+^) per well. Subsequently, 50 µl of assay reagent were added following vigorous mixing for further promoting penetration of the 3D reagent into the spheroid matrix. The plate was incubated in the dark for 25 min at room temperature and luminescence was read using a SpectraMax Gemini XS (Molecular Devices, San Jose, CA, USA) reader.

### Triglyceride imaging

Intracellular neutral lipid droplets were stained using Nile Red staining, nuclei were stained using Hoechst 33342 (Invitrogen, Carlsbad, CA). In brief, a double-staining solution containing 2 µM Nile Red and 16 µM Hoechst 33342 was prepared in PBS (w/o Mg^2+^, Ca^2+^). Prior to staining, spheroids were fixed in a 4% paraformaldehyde solution at room temperature for 1 hour, followed by storage at 4 °C in PBS (w/o Mg^2+^, Ca^2+^). Fixed spheroids were incubated with double-staining solution at room temperature for 90 min, followed by incubation at 37 °C for 30 min and washing with PBS (w/o Mg^2+^, Ca^2+^). Confocal imaging was performed in a black 96-well assay plate (353219 Corning, Corning, NY, USA) using an LSM880 Airyscan (Zeiss, Jena, Germany) microscope.

### Intracellular triglyceride quantification

Intracellular TG content was determined using a fluorescent probe that specifically partitions into fat droplets (AdipoRed, Lonza, Switzerland), following the manufacturer’s instructions with slight modifications. For facilitating access of the reagent to all cells, the spheroids were disintegrated using trypsin treatment. Trypsin solution was prepared by a 1:1 dilution 0.5% trypsin-EDTA with PBS (w/o Mg^2+^, Ca^2+^), of which 200 µl were used per assay in a black 96-well assay plate (3631 Corning, Corning, NY, USA). For each spheroid replicate (total of 16 per time point), one individual spheroid was taken up in 20 µl of its maintenance or treatment medium, respectively, and the complete volume transferred into the trypsin solution. For determining the culture medium-derived background signal, for each replicate (total of eight per time point) 20 µl of maintenance or treatment medium supernatant, respectively, without spheroid were added to the trypsin solution. The fully prepared assay plate was incubated at 37 °C for 10 min. Following, 7 µl of AdipoRed reagent was added to each replicate of spheroid sample and culture medium background assay, respectively. For full dissociation of the 3D spheroid cell aggregates, repeated and vigorous resuspending of the suspension was performed. Subsequently, the assay plate was incubated in the dark at room temperature for 10 min and fluorescence intensity was measured (ex: 485 nm, em: 572 nm) on an infinite 200 reader (Tecan, Männedorf, Switzerland). The culture medium-derived signal for each condition was subtracted from all respective spheroid samples prior to determining the relative fold change of fluorescence signal intensity between FA-free BSA control and FA-incubated conditions.

### Intracellular protein isolation

For protein isolation, spheroids were pooled and washed twice with PBS (w/o Mg^2+^, Ca^2+^). Cell lysis was accomplished by repeated vigorous resuspension of the spheroids at 4 °C in a mixture of M-PER Mammalian Protein Extraction Reagent (Thermo Scientific, Rockford, IL, USA) and cOmplete^TM^ Mini Protease Inhibitor (Roche, Mannheim, Germany) working solution (prepared according to the manufacturer’s instructions), in a ratio of 9:1. The homogenate was sonicated 5 times for 15 s in ice cold water and subsequently centrifuged at 12000 × *g* and 4 °C for 15 min. The supernatant was extracted and total protein concentration determined on a Nanodrop 1000 spectrophotometer (NanoDrop Technologies, Wilmington, DE, USA). Samples were stored at −80 °C until further analysis.

### Immunoblotting for APOB detection

Cell media samples were concentrated 10 times using Vivaspin 500 columns (Sartorius Stedim Lab Ltd, Stonehouse, UK) for the APOB-100 detection. Cell lysates as well as concentrated and diluted cell media samples were mixed with Laemmli buffer containing 2-mercaptoethanol (ratio sample: Laemmli buffer = 4:1 v/v) and incubated for 5 min at 95 °C. Proteins were size-separated by SDS-page (6% acrylamide gel, 90 V, 3 h at 4 °C for APOB-100 and calnexin detection; 10% acrylamide gel, 130 V, 1 h at room temperature for albumin detection) and transferred onto a nitrocellulose membrane (0.2 A, 2.5 h at 4 °C). Nitrocellulose membranes were incubated overnight with primary antibodies, washed 3 times for 10 min with tris-buffered saline containing 0.2% tween (0.2% TBS-T buffer), incubated 1 h with HRP-conjugated secondary antibodies, washed 3 times for 10 min with 0.2% TBS-T buffer, incubated with Immobilon Western chemiluminescent HRP substrate (Millipore Corporation, Billerica, Massachusetts, USA). Bands were visualized using Chemidoc XRS System (Biorad, Hercules, California, USA). The following antibodies were used: mouse anti-APOB (Santa Cruz Biotechnology, Dallas, Texas, USA), rabbit anti-calnexin (Sigma-Aldrich, Saint Louis, Missouri, USA), mouse anti-albumin (Sigma-Aldrich, Saint Louis, Missouri, USA).

### Genotyping

DNA was extracted from hepatocytes using DNeasy Blood & Tissue Kit (Qiagen, Hilden, Germany). DNA from all donors was undergone to genotyping for *TM6SF2*_rs58542926 (E167K), PNPLA3 rs738409 (I148M), PNPLA3 rs2294918 (E434K), GCKR rs1260326 (P446L) and MBOAT7 rs641738 (C > T) polymorphisms by TaqMan™ assays (Life Technologies, Carlsbad, CA, USA) using CFX384 Real Time PCR detection system (Bio-Rad Laboratories, Hercules, CA, USA). Data analysis was performed using Bio-Rad CFX manager software.

### Gene expression analysis

Total RNA was isolated using miRNeasy Mini Kit (Qiagen, Hilden, Germany). RNA was reverse-transcribed into cDNA using High Capacity cDNA Reverse Transcription (Applied Biosystems, Waltham, MA, USA). RT-qPCR analysis was performed on customized microfluidic TaqMan™ array 384-well cards using TaqMan™ Fast Advanced Master Mix (Applied Biosystems, Waltham, MA, USA) on a QuantStudio^TM^ 7 Flex Real-Time PCR System (Applied Biosystems, Waltham, MA, USA). QuantStudio^TM^ Real-Time PCR Software, version 1.3, (Applied Biosystems, Waltham, MA, USA) was used for data analysis.

## Supplementary information

Supplementary Information

Supplementary Table S2

Supplementary Table S3

Supplementary Table S4

## Data Availability

The datasets generated during and/or analysed during the current study are available from the corresponding author on reasonable request.
