# Supplementary material for: The TM6SF2 E167K genetic variant induces lipid biosynthesis and reduces apolipoprotein B secretion in human hepatic 3D spheroids
Source: Sci Rep. 2019 Aug 12;9:11585. doi: 10.1038/s41598-019-47737-w (PMC6690969; doi:10.1038/s41598-019-47737-w)
Supplement: Supplementary file 1 — Supplementary Information [file 41598_2019_47737_MOESM1_ESM.docx]

# **Supplementary Information | SREP-18-47775**

**The TM6SF2 E167K genetic variant induces lipid biosynthesis and reduces apolipoprotein B secretion in human hepatic 3D spheroids**

Sebastian Prill^1^, Andrea Caddeo^2^, Guido Baselli^3^, Oveis Jamialahmadi^2^, Paola Dongiovanni^3^, Raffaela Rametta^3^, Kajsa P Kanebratt^1^, Arturo Pujia^4^, Piero Pingitore^2^, Rosellina Margherita Mancina^2^, Daniel Lindén^5,6^, Carl Whatling^7^, Annika Janefeldt^1^, Mikael Kozyra^8^, Magnus Ingelman-Sundberg^8^, Luca Valenti^3^, Tommy B Andersson^1, 8^ & Stefano Romeo^2, 4, 9*^

*^1^DMPK, Cardiovascular, Renal and Metabolism, IMED Biotech Unit, AstraZeneca, Gothenburg, Sweden
^2^Department of Molecular and Clinical Medicine, University of Gothenburg, Sweden
^3^Internal Medicine and Metabolic Diseases, Fondazione IRCCS Ca’ Granda Ospedale Maggiore Policlinico Milano, Department of Pathophysiology and Transplantation, Università degli Studi di Milano, Milan, Italy
^4^Clinical Nutrition Unit, Department of Medical and Surgical Sciences, University Magna Graecia, Catanzaro, Italy
^5^Bioscience Diabetes, Cardiovascular, Renal and Metabolism, IMED Biotech Unit, AstraZeneca, Gothenburg, Sweden
^6^Division of Endocrinology, Department of Neuroscience and Physiology, Sahlgrenska Academy, University of Gothenburg, Sweden
^7^ Translational Sciences, Cardiovascular, Renal and Metabolism, IMED Biotech Unit, AstraZeneca, Gothenburg, Sweden
^8^Department of Physiology and Pharmacology, Section of Pharmacogenetics, Karolinska Institutet, Stockholm, Sweden
^9^Cardiology Department, Sahlgrenska University Hospital, Gothenburg, Sweden*

**To whom correspondence should be addressed: Department of Molecular and Clinical Medicine at Sahlgrenska University Hospital, Bruna Stråket 13, 41345 Gothenburg, Sweden; Tel:* *+46313422929; Email: stefano.romeo@wlab.gu.se*

**SUPPLEMENTARY TABLE S1:** Clinical characteristics of patients. Data are presented as mean ± SD. (): % values, {}: interquartile range. Abbreviations: BMI, body max index; NAS, nonalcoholic fatty liver disease activity score; LDL, low-density lipoprotein; HDL, high-density lipoprotein; PNPLA3, patatin-like phospholipase domain-containing protein 3.

|  | ***TM6SF2***  **rs58542926 CC**  **(N=116)** | ***TM6SF2***  **rs58542926 CT**  **(N=9)** | **p-value** |
| --- | --- | --- | --- |
| Age | 44±10 | 34±10 | 0.005 |
| Sex, F | 100 (86) | 7 (78) | 0.841 |
| BMI, Kg/m^2^ | 41±7 | 42±8 | 0.495 |
| Diabetes, Yes | 14 (12) | 1 (11) | 0.999 |
| Steatosis, Yes | 97 (84) | 7 (78) | 0.999 |
| Fibrosis, Yes | 40 (34.8) | 2 (22.2) | 0.688 |
| NAS > 3, Yes | 13 (11) | 1 (11) | 0.999 |
| ALT, U/L | 20 {15-29} | 22.00 {15.0-28.0} | 0.740 |
| AST, U/L | 18 {15-23} | 19.00 {16-20} | 0.831 |
| Cholesterol, mg/dl | 209±47 | 199±39 | 0.658 |
| LDL, mg/dl | 133±38 | 115±8 | 0.328 |
| HDL, mg/dl | 55±14 | 55±11 | 0.993 |
| Triglycerides, mg/dl | 118 {88-164} | 132 {91-17} | 0.809 |
| PNPLA3 I148M |  |  | 0.666 |
| I/I | 55 (47) | 5 (56) |  |
| I/M | 52 (45) | 4 (44) |  |
| M/M | 9 (8) | 0 (0) |  |

**SUPPLEMENTARY TABLE S2:** RNAseq data. Results of differential gene expression analysis performed by DESeq2 data analysis pipeline, including fold change expression and the nominal p-values. See Excel file “Supplementary Table S2.XLS”

(Double click to open)

**SUPPLEMENTARY TABLE S3:** Spheroid gene expression data. Expression levels of genes in the cholesterol, FA metabolism and gluconeogenesis pathway from two TM6SF2 E167K and three wild type donors. See Excel file “Supplementary Table S3.xlsx”

(Double click to open)

**SUPPLEMENTARY TABLE S4:** Gene Ontology (GO) biological process (BP) terms and KEGG biological pathways. See Excel file “Supplementary Table S4.XLS”

(Double click to open)

**SUPPLEMENTARY TABLE S5:** Sourcing details and demographics of primary human hepatocyte donors. Abbreviations: BMI, body max index; *TM6SF2*, transmembrane 6 superfamily member 2; PNPLA3, patatin-like phospholipase domain-containing protein 3; GCKR, glucokinase regulatory protein; MBOAT7, membrane bound O-acyltransferase domain containing 7; TM6-1, *TM6SF2* E167K-1; TM6-2, *TM6SF2* E167K-2

| **Donor** | **Sex** | **Age** | **BMI** | **Cause of death** | ***TM6SF2* E167K**  **rs58542926** | **PNPLA3 I148M**  **rs738409** | **PNPLA3 E434K**  **rs2294918** | **GCKR P446L**  **rs1260326** | **MBOAT7 C>T**  **rs641738** |
| --- | --- | --- | --- | --- | --- | --- | --- | --- | --- |
| **TM6-1** | M | 22 | 22.4 | Cerebral hemorr. | CT | CC | GA | CC | CC |
| **TM6-2** | F | 13 | 32.1 | Head Trauma | CT | CC | GA | CC | CT |
| **WT-1** | M | 44 | 23.4 | Anoxia | CC | CC | GA | CC | TT |
| **WT-2** | F | 30 | 30.8 | Head Trauma | CC | CC | GG | CT | CT |
| **WT-3** | F | 78 | 26.8 | Stroke | CC | CC | GA | CT | CT |


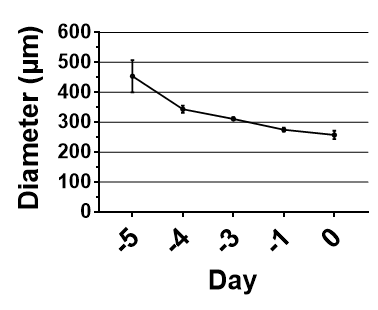


**SUPPLEMENTARY FIGURE S1: Spheroids condense during formation.** Cell aggregate diameter was measured during spheroid formation. During that period, diameter decreased by approximately 45%. Representative data from the WT-1 donor. Values show the mean ± SD from 4 individual spheroid replicates per day, and of one spheroid preparation. Abbreviation: SD, standard deviation; WT, wild type.


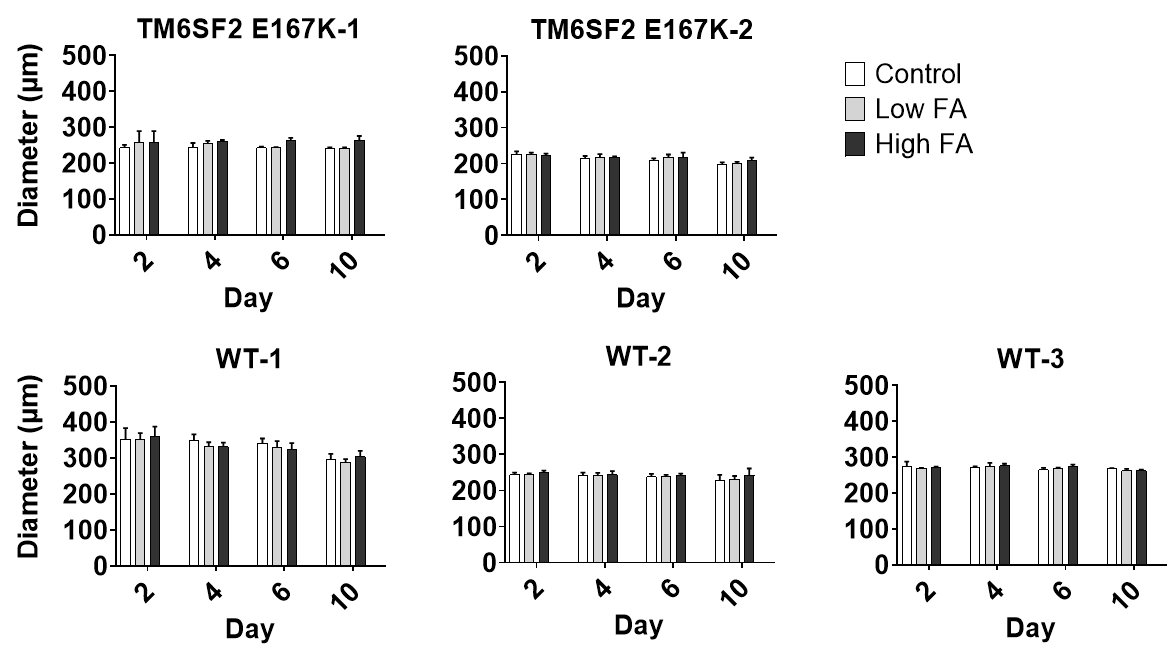


**FIGURE S2: Spheroids maintain diameter throughout culture and fatty acid incubation time.** Spheroid diameter was determined to be consistent throughout the experiment period of 10 days, with and without incubation with FA, across the two *TM6SF2* E167K donors and three WT donors. Data represent mean values ± SD from 5 individual spheroid replicates per condition and day, and of one spheroid preparation per donor. Abbreviations: FA, fatty acid; WT, wild type.


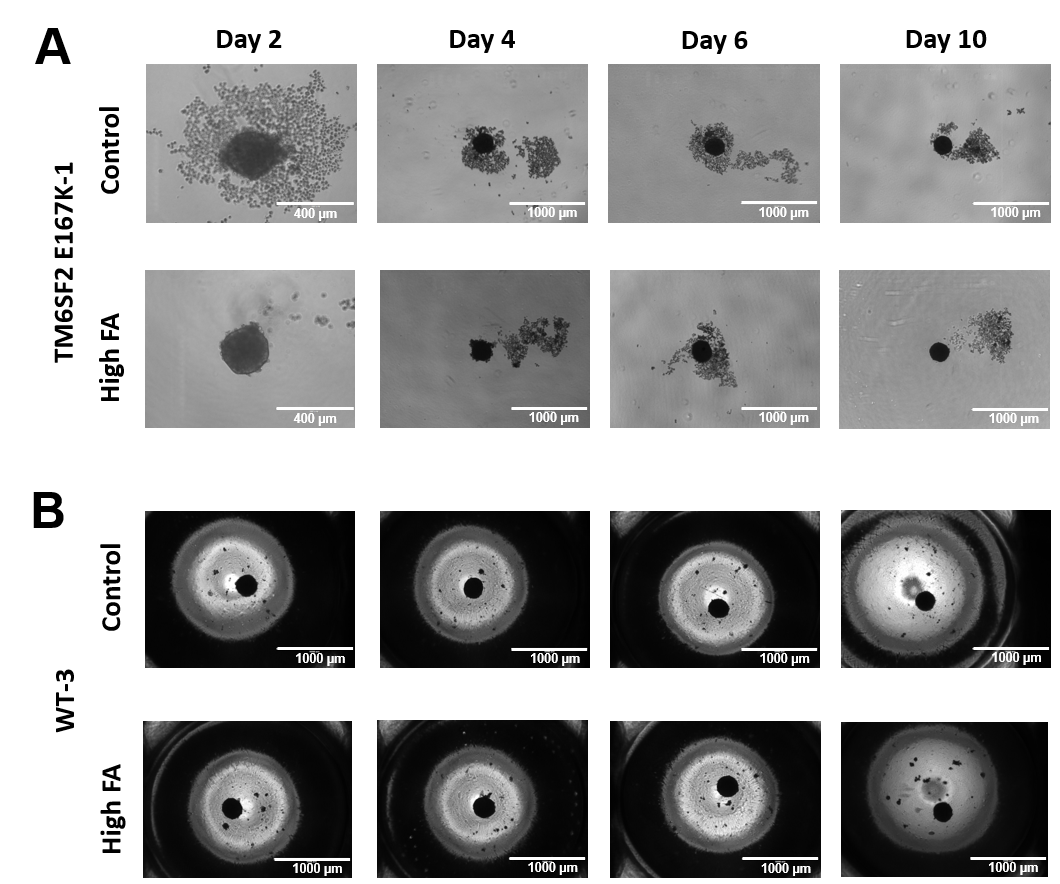


**FIGURE S3: Spheroids show no morphological changes for up to 10 days of FA treatment.** (A,B) Bright-field images of spheroids from the *TM6SF2* E167K-1 donor (A) and the WT-3 donor (B), without (top row, Control, FA-free BSA) and with FA treatment (bottom row, high FA, BSA + 213 µM OA / 107 µM PA) for up to 10 days showed no morphological differences between the two populations. Non-integrated, likely not viable cells primarily visible in the *TM6SF2* E167K-1 preparation can be ascribed to the fact that cell viabilities across donors varied at seeding. 2000 viable cells were seeded in all preparations, yielding equally sized spheroids. Abbreviations: FA, fatty acid; OA, oleic acid; PA, palmitic acid; WT, wild type.


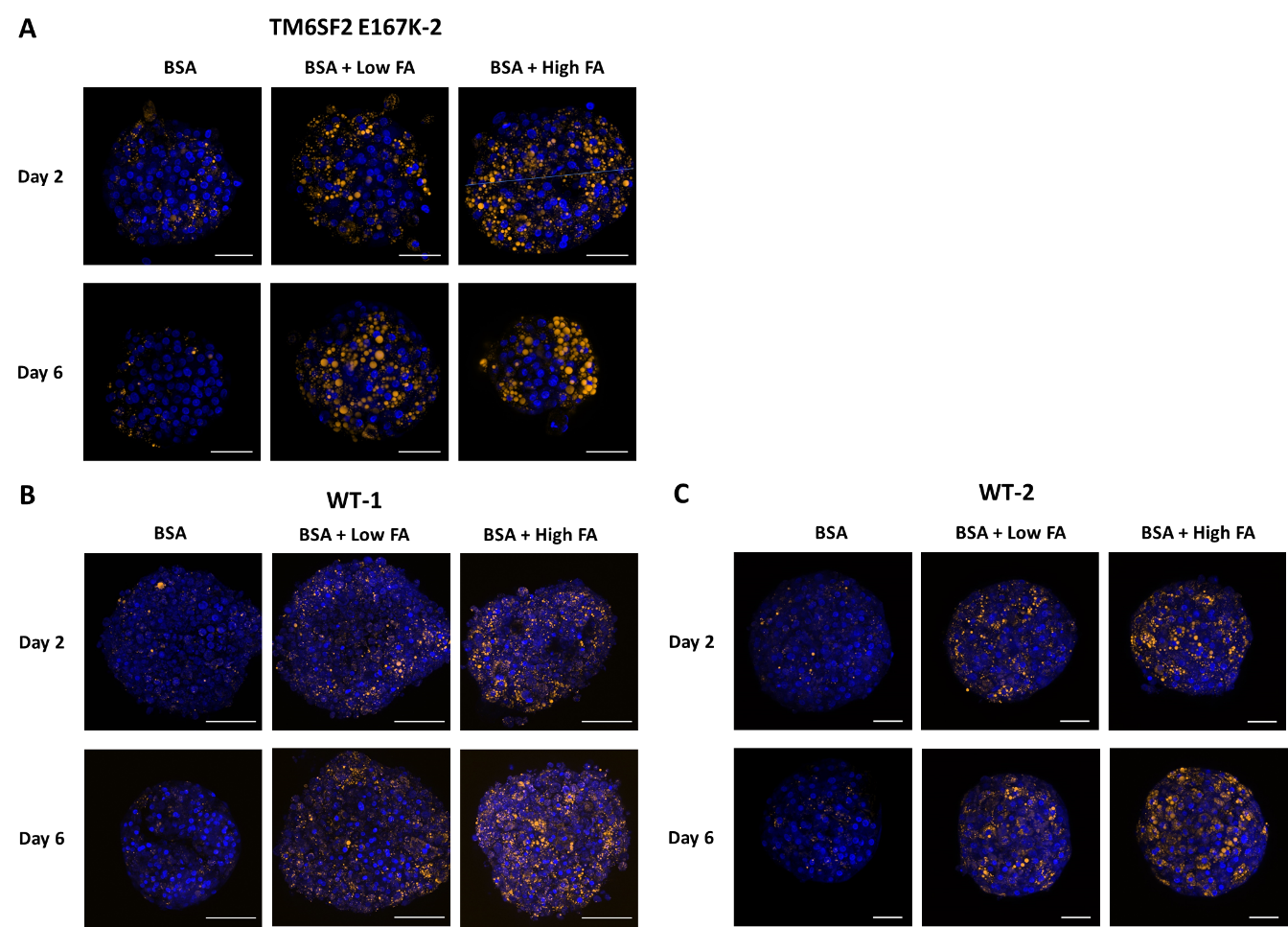


**FIGURE S4: Nile red + Hoechst staining of spheroids from primary human hepatocytes of two individual donors after fatty acid treatment for 2 and 6 days.** (A-C) Nile red staining of liver spheroids derived from three individual hepatocyte donors, treated with a 2:1 mixture of OA and PA. Spheroids were exposed to low (107 µM OA / 53 µM PA) and high (213 µM OA / 107 µM PA) concentrations of FAs. The *TM6SF2* E167K-2 donor was heterozygous for the Transmembrane 6 superfamily member 2 (*TM6SF2*) sequence variant (rs58542926 C>T) encoding for E167K aminoacidic substitution, both other donors were WT for the variant in *TM6SF2*. Abbreviations: FA, fatty acid; OA, oleic acid; PA, palmitic acid; WT, wild type.


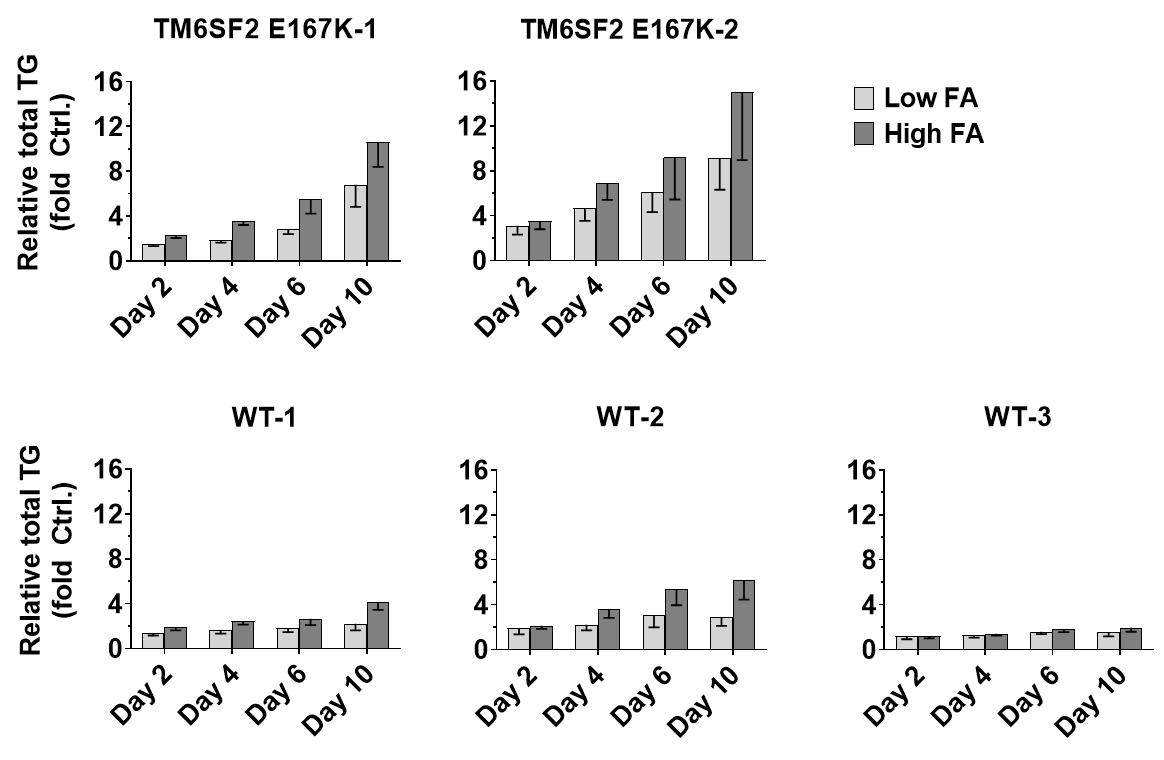


**FIGURE S5: Relationship between three fatty acid concentrations and intracellular fat accumulation.** Incubation with low (BSA + 107 µM OA / 53 µM PA) and high (BSA + 213 µM OA / 107 µM PA) FA concentration resulted in a FA concentration-dependent increase in intracellular TG. TG content is shown as fold change relative to vehicle control (FA-free BSA). Abbreviations: BSA, bovine serum albumin; FA, fatty acid; OA, oleic acid; PA, palmitic acid; TG, triglyceride


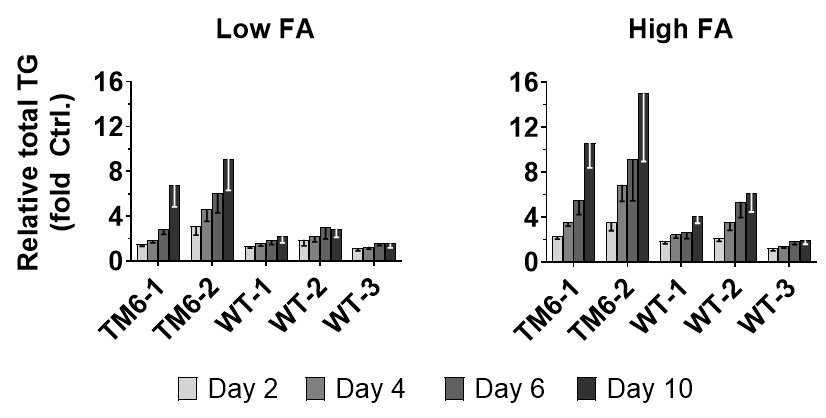


**FIGURE S6: Relationship between incubation time with fatty acids and intracellular fat accumulation.** Incubation over 10 days, with samplings on days 2, 4, 6 and 10, resulted in a time-dependent increase in intracellular TG for low (BSA + 107 µM OA / 53 µM PA) and high (BSA + 213 µM OA / 107 µM PA) FA concentration. TG content is shown as fold change relative to vehicle control (FA-free BSA). Abbreviations: BSA, bovine serum albumin; FA, fatty acid; OA, oleic acid; PA, palmitic acid; TG, triglyceride; TM6-1, TM6SF2 E167K-1; TM6-2, TM6SF2 E167K-2


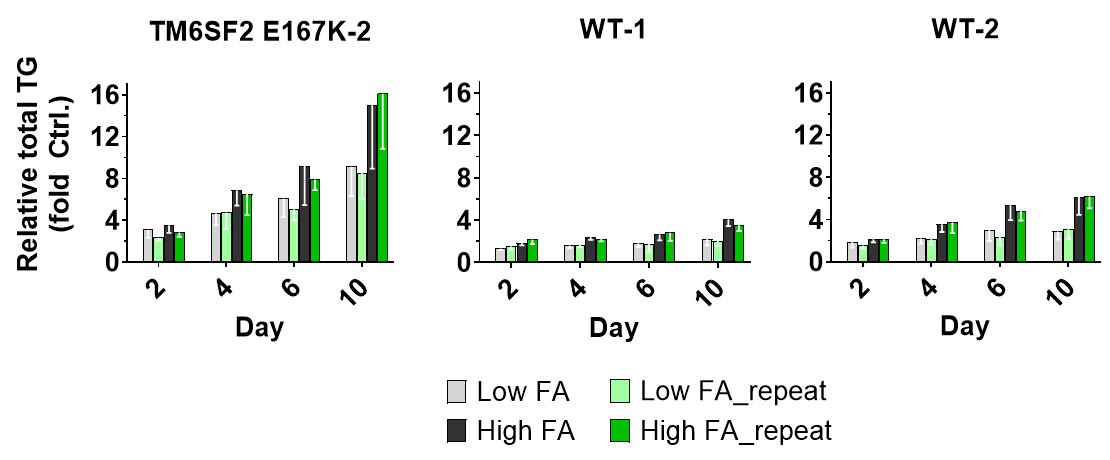


**FIGURE S7: Liver spheroids from three donors (TM6SF2 E167K-2, WT-1, WT-2) robustly reproduce the donor-specific patterns of total TG accumulation in repeat experiments using fatty acid incubation.** For each of the shown donors, one additional spheroid preparation was performed at a later time point, with subsequent FA-treatment as shown and described in Figure 4, using low (107 µM OA/53 µM PA) and high (213 µM OA/107 µM PA) FA concentration (green bars). Abbreviations: FA, fatty acid; OA, oleic acid; PA, palmitic acid; TG, triglyceride; WT, wild type.


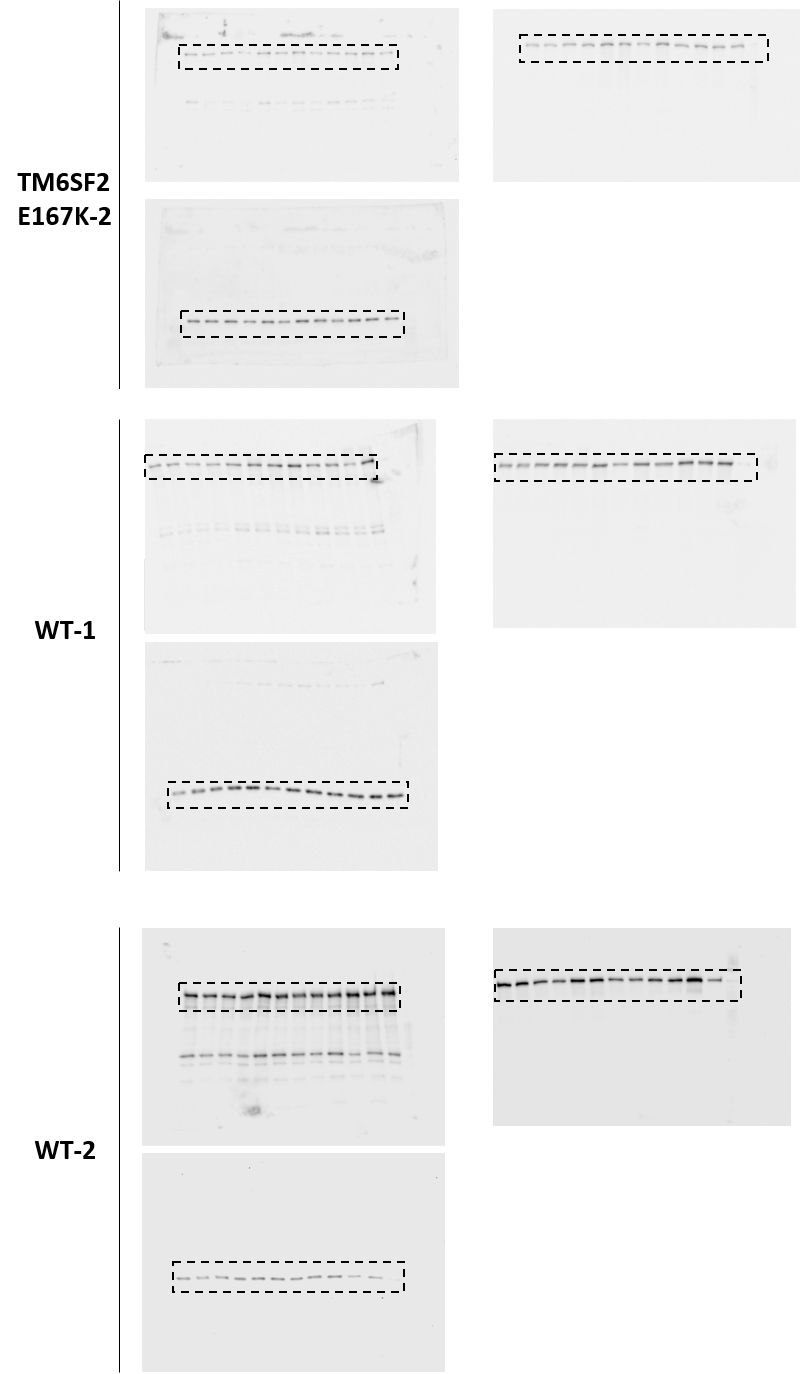


**FIGURE S8: Uncropped images of blots used for Figure 4.** Cropped areas that have been used for compliling Figure 4 are boxed in dashed lines. The layout of single gels from the original Figure 4 is maintained.


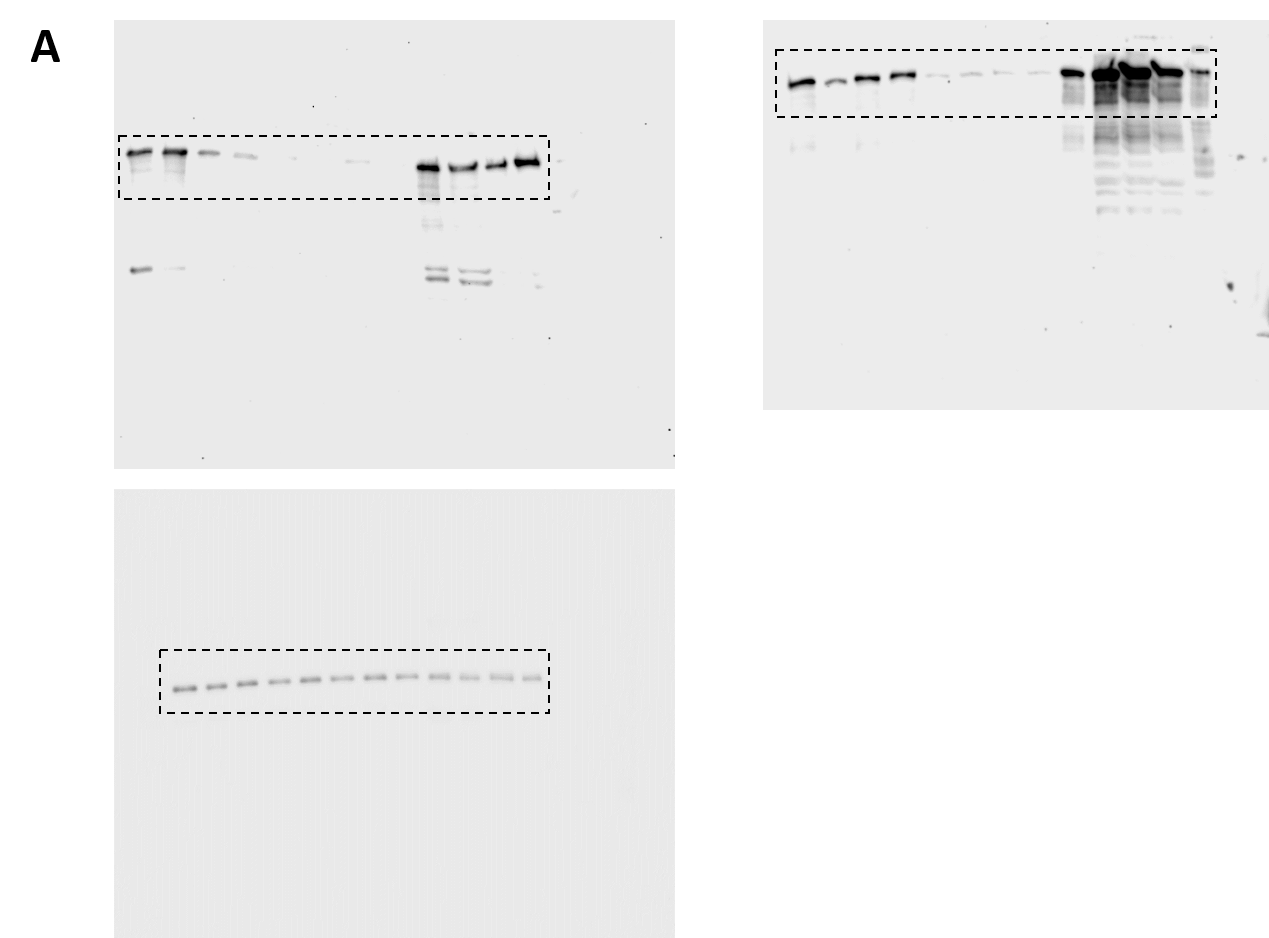


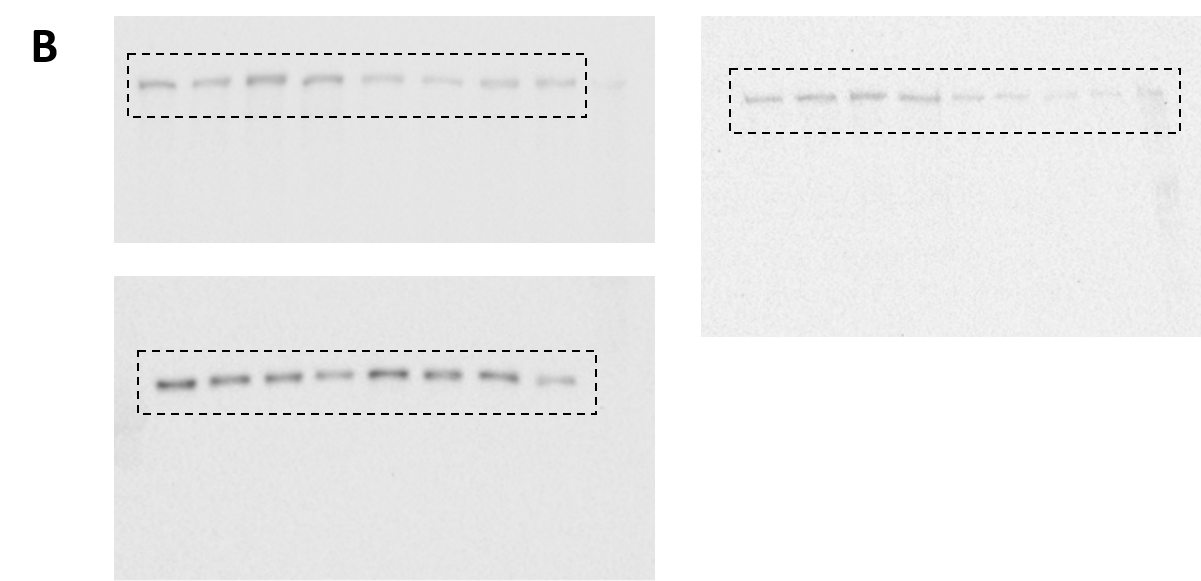


**FIGURE S9: Uncropped images of blots used for Figure 7.** (A) Cropped areas that have been used for compliling Figure 7A are boxed in dashed lines. (B) Cropped areas that have been used for compliling Figure 7B are boxed in dashed lines. The layout of single gels from the original Figure 7A and 7B is maintained.
